# Association between body stature with ocular biometrics and refraction among Chinese preschoolers

**DOI:** 10.1186/s12886-024-03372-2

**Published:** 2024-03-06

**Authors:** Linling Li, Chimei Liao, Xiaojuan Zhang, Juan Lu, Yangfa Zeng, Min Fu, Decai Wang

**Affiliations:** 1 Shenzhen Maternity and Child Healthcare Hospital, No. 2004, Hongli Road, Futian district, 510060 Shenzhen, People’s Republic of China; 2 Zhongshan Ophthalmic Center, State Key Laboratory of Ophthalmology, Sun Yat-Sen University, 54S.Xianlie Road, 510060 Guangzhou, People’s Republic of China

**Keywords:** Association, Body stature, Ocular biometrics, Refraction, Preschool children

## Abstract

**Purpose:**

To evaluate the association of body stature with ocular biometrics and refraction in preschool children.

**Methods:**

A cross-sectional, school-based study was conducted in Shenzhen, China. Preschool children aged 3 to 6 from 10 randomly-selected kindergartens were recruited. Ocular biometric parameters, including axial length (AL), anterior chamber depth (ACD), vitreous chamber depth (VCD), corneal radius curvature (CR), axial length to corneal radius ratio (AL-to-CR ratio) and lens thickness (LT) were measured using non-contact partial-coherence laser interferometry. Cycloplegic refractions were obtained by a desktop autorefractor. Body height and weight were measured using standard procedures. The association between body stature and ocular biometrics were analyzed with univariable and multivariable regression model.

**Results:**

A total of 373 preschoolers were included. AL, ACD, VCD, CR, and AL-to-CR ratio, were positively associated with height and weight (*p* < 0.05), whereas LT was negatively associated with height and weight (*p* < 0.01). No association was observed between stature and central cornea thickness and refraction. After adjusted for age and gender in a multivariable regression model, AL had positive associations with height (*p* < 0.01) and weight (*p* < 0.01). However, refraction had no significant association with stature parameters.

**Conclusion:**

Taller and heavier preschoolers had eyes with longer AL, deeper vitreous chamber, and flatter cornea. The significant associations between body stature and ocular biometric parameters reveal the driving influence of body development on the growth of eyeballs in preschoolers.

## Introduction

Myopia is a highly prevalent ocular disorder globally, especially in Asia [1]. Uncorrected visual acuity loss caused by myopia is the most common reason for visual impairment worldwide [2]. As the prevalence is yet increasing, it’s posing a major public health challenge.

Myopia occurs when there is a mismatch between the focal plane of the eye and the prolonged axial length (AL). During the postnatal eye growth, proportional changes in the ocular biometric parameters such as AL, anterior chamber depth (ACD), lens thickness (LT), corneal curvature (CR), and vitreous chamber depth (VCD) bring the eye to emmetropization and the disturbance of this highly coordinated process will lead to refractive errors [3]. Thus, a thorough understanding of the optical and structural development of the eye during emmetropization and its related factors helps to reveal the potential mechanism of myopia incidence.

It’s believed that a shared mechanism exists for ocular development and body stature since the eye grows at a time when body stature is also increasing. Previous studies have indicated that AL is associated with height, weight, and body mass index (BMI) and that longitudinal changes in AL and height are concomitant in children [4]. Twin study provided evidence that 89% of the phenotypic correlation of AL and height was due to shared genetic factors [5]. However, inconsistent results were observed [6–11]. For instance, the Anyang University Students Eye Study found a negative association between height and refraction [6], whereas Tien-Yin Wong et al. demonstrated no association between height and refraction [7]. Most previous studies have primarily included children older than seven years or adults, among which period myopia progression is susceptible to education and other confounding factors. A few studies have described the relationship between body stature and refraction, AL or CR in preschool children [12–14], but its relationship with other ocular biometrics was rarely discussed.

In this study, we aim to describe the distribution of refraction and comprehensive ocular biometrics including AL, central corneal thickness (CCT), ACD, LT, VCD, CR and AL-to-CR ratio, as well as to investigate their relationship with body stature (height, weight, BMI) in a preschool population from Shenzhen city, southern China.

## Methods

### Study population

This study is a school-based, cross-sectional study conducted in a group of preschoolers in Futian District, Shenzhen, Guangdong Province in southern China.

Participants were recruited from 10 randomly selected kindergartens with facilities and teaching staff of the same level. The study followed the tenets of the Declaration of Helsinki. Ethics approval was obtained from the Institutional Review Board of Shenzhen Maternity and Child Healthcare Hospital (SFYLS [2020] 042). The purpose and examination procedures of the study were explained to the parents or legal guardians at the ophthalmic clinic of Shenzhen Maternity and Child Healthcare Hospital, after which written informed consent was obtained.

### Inclusion and exclusion criteria

Four hundred and nineteen preschoolers aged 3 to 6 years were enumerated and have given informed consent for participation. Children were excluded as they had congenital diseases, developmental disabilities, systemic diseases, eye diseases, and other diseases that would affect the development of stature or ocular biometry. Those failed to finish all the examinations were also excluded. As a result, three hundred and seventy-three preschoolers were included into final analysis.

### Examinations

Ocular examinations were conducted at the ophthalmic clinic of Shenzhen Maternity and Child Healthcare Hospital from September to October, 2020. Ocular biometrics, including AL, ACD and LT were measured before pupil dilation with a non-contact partial-coherence laser interferometry (IOL Master; Carl Zeiss Meditec, Oberkochen, Germany). After intraocular pressure measurement with a non-contact tonometer (CT-1, Topcon, Japan), 1% cyclopentolate drops were administered to participants with normal intraocular pressure to induce cycloplegia. After the pupils were fully dilated, refraction examinations were taken using a desktop autorefractor (KR8800; Topcon Corp., Tokyo, Japan). Height and weight were measured without shoes in a standardized manner.

### Definitions

Spherical equivalent refraction (SER) was calculated as spherical diopters (D) plus one-half cylindrical diopters using data extracted from the autorefractor. Corneal radius (CR) was calculated as the mean of the greatest CR and the lowest one. Vitreous chamber depth (VCD) was calculated as the AL minus ACD and LT. The AL-to-CR ratio was computed as AL in millimeters divided by CR in millimeters. BMI was computed using the formula BMI = weight(kg)/height(m)^2^.

### Statistics

The distribution of ocular biometrics, including AL, ACD, LT, VCD, CR, AL-to-CR ratio, refraction presented as SER, and stature parameters, including height, weight, and BMI, were presented and compared by gender using t-tests. As the ocular biometrics and refraction of both eyes are highly correlated (Pearson’s correlation coefficient for AL, 0.98; CCT, 0.98; ACD, 0.98; LT, 0.99; VCD, 0.97; CR, 0.97; AL-to-CR ratio, 0.96, and SER, 0.89), only the data of the right eyes were presented and analyzed. Mean, standard deviation and the range of body stature, ocular biometrics, and refraction were presented. Trend analysis of ocular biometrics and refraction was performed by height, weight, and BMI quartiles to detect a significant difference. Univariable analyses were performed to determine the associations of height, weight, and BMI with different ocular biometric components and refraction. Multivariable regression analyses adjusted by age and gender were performed to assess the effects of height, weight, and BMI on ocular biometrics and refraction. In the regression model, individual refraction or ocular biometric components were analyzed as the dependent variables, and height, weight, or BMI were the independent variables. A two-sided P value of 0.05 or less was considered statistically significant. All analyses were performed using the Stata Statistical Software (version 16.0; Stata Corp, College Station, TX). All confidence intervals (CIs) are 95%.

## Results

A total of 373 children were enrolled in the analysis, with a mean age of 4.91 ± 0.79 years without statistically significant gender difference (t = 1.62, *p* = 0.11). In general, girls were shorter (109.26 ± 7.11 cm vs. 111.71 ± 6.74 cm, t = 3.41, *p* < 0.01) and lighter (18.32 ± 3.16 kg vs. 19.32 ± 2.81 kg, t = 3.23, *p* < 0.01) than boys (Table 1). The average SER of the population was 1.33 ± 0.79D, and greater hyperopia was observed in girls (1.48 ± 0.84, t=-3.71, *p* < 0.01) with shorter AL (21.97 mm vs. 22.57 mm, t = 9.35, *p* < 0.01), smaller CCT (0.54 mm vs. 0.55 mm, t = 3.54, *p* < 0.01) and CR (7.71 mm vs., 7.83 mm, t = 5.15, *p* < 0.01), shallower ACD (3.43 mm vs. 3.56 mm, t = 6.29, *p* < 0.01), smaller VCD (14.44 mm vs. 14.94 mm, t = 8.01, *p* < 0.01) and thicker LT (3.56 mm vs. 3.52 mm, t=-2.95, *p* < 0.01).

**Table 1:** Means of age, ocular biometrics and stature measurements by gender group

	Total				Male			Female				
Variables	N	Mean ± SD	Range(Min ~ Max)	N		Mean ± SD	Range(Min ~ Max)	N	Mean ± SD	Range(Min ~ Max)	t-value	*P*-value
**Age(years)**	373	4.91 ± 0.79	3.02 ~ 6.97	182		4.98 ± 0.79	3.28 ~ 6.86	191	4.85 ± 0.79	3.02 ~ 6.97	1.62	0.11
**Height(cm)**	373	110.45 ± 7.03	87.00 ~ 130.00	182		111.71 ± 6.74	96.50 ~ 130.00	191	109.26 ± 7.11	87.00 ~ 127.00	3.41	< 0.01
**Weight(kg)**	373	18.81 ± 3.03	11.50 ~ 27.50	182		19.32 ± 2.81	13.00 ~ 27.00	191	18.32 ± 3.16	11.50 ~ 27.50	3.23	< 0.01
**BMI(kg/m** ^**2**^ **)**	373	15.36 ± 1.58	8.06 ~ 20.74	182		15.44 ± 1.43	11.57 ~ 19.95	191	15.29 ± 1.71	8.06 ~ 20.74	0.96	0.34
**SER(D)**	373	1.33 ± 0.79	-2.00 ~ 4.75	182		1.18 ± 0.71	-1.13 ~ 3.88	191	1.48 ± 0.84	-2.00 ~ 4.75	-3.71	< 0.01
**AL(mm)**	373	22.27 ± 0.69	19.90 ~ 24.69	182		22.57 ± 0.60	20.94 ~ 24.69	191	21.97 ± 0.64	19.90 ~ 23.45	9.35	< 0.01
**CCT(mm)**	373	0.55 ± 0.03	0.47 ~ 0.62	182		0.55 ± 0.03	0.47 ~ 0.62	191	0.54 ± 0.03	0.47 ~ 0.62	3.54	< 0.01
**ACD(mm)**	373	3.49 ± 0.21	2.79 ~ 4.09	182		3.56 ± 0.20	3.05 ~ 4.09	191	3.43 ± 0.19	2.79 ~ 4.02	6.29	< 0.01
**LT(mm)**	373	3.54 ± 0.14	3.12 ~ 3.92	182		3.52 ± 0.15	3.19 ~ 3.90	191	3.56 ± 0.14	3.12 ~ 3.92	-2.95	< 0.01
**VCD(mm)**	373	14.68 ± 0.65	12.46 ~ 17.04	182		14.94 ± 0.59	13.51 ~ 17.04	191	14.44 ± 0.61	12.46 ~ 15.75	8.10	< 0.01
**CR(mm)**	360	7.77 ± 0.23	7.25 ~ 8.61	175		7.83 ± 0.22	7.25 ~ 8.44	185	7.71 ± 0.22	7.31 ~ 8.61	5.15	< 0.01
**AL-to-CR ratio**	360	2.87 ± 0.07	2.64 ~ 3.09	175		2.88 ± 0.07	2.74 ~ 3.09	185	2.85 ± 0.07	2.64 ~ 3.04	4.72	< 0.01

SD = standard deviation,AL = axial length, CCT = central cornea thickness, ACD = anterior chamber depth, LT = Lens thickness, VCD = vitreous chamber depth, CR = corneal radius curvature, AL-to-CR = axial length to corneal radius, SER = spherical equivalent refraction

p based on t-test

The correlations of height, weight, and BMI, with ocular biometric parameters and SER, are shown in Table 2. Only height was correlated with SER (*r*=-0.12, *p* < 0.05). Both height and weight were positively and significantly correlated with AL (*r* = 0.36 and 0.34), ACD (*r* = 0.18 and 0.19), VCD (*r* = 0.39 and 0.34), CR (*r* = 0.12 and 0.17), and AL-to-CR ratio (*r* = 0.31 and 0.21) but negatively correlated with LT (*r*=-0.32 and − 0.23, specifically). BMI had no correlation with refraction and biometrics except for CR (*r* = 0.12, *p* < 0.05). There was no significant correlation between body stature and CCT (all *r* < 0.1 and *p* > 0.05 ).

**Table 2:** Correlation analysis between stature and refraction, ocular biometry

	correlation coefficients		
Variables	Height(cm)	Weight(kg)	BMI(kg/m^2^)
SER (D)	-0.12*	-0.08	0.02
AL (mm)	0.36**	0.34**	0.09
CCT (mm)	0.01	0.03	0.05
ACD (mm)	0.18**	0.19**	0.08
LT (mm)	-0.32**	-0.23**	0.04
VCD (mm)	0.39**	0.34**	0.05
CR (mm)	0.12*	0.17**	0.12*
AL-to-CR ratio	0.31**	0.21**	-0.05

SD = standard deviation,AL = axial length, CCT = central cornea thickness, ACD = anterior chamber depth, LT = Lens thickness, VCD = vitreous chamber depth, CR = corneal radius curvature, AL-to-CR = axial length to corneal radius, SER = spherical equivalent refraction

P based on Pearson correlation test, ** *p* < 0.01, * *p* < 0.05

The mean values of refraction and ocular biometrics of the children, categorized by quartiles of height, weight, and BMI, are shown in Table 3. Height and weight quartiles were significantly associated with ocular biometrics except for CCT. Generally, higher and heavier children tended to have longer eyes, deeper ACD, thinner lenses, deeper VCD, flatter cornea and larger AL-to-CR ratio (all *p* < 0.05). By contrast, refraction didn’t change significantly with increasing height and weight. Children with higher BMI values tended to have flatter cornea, while other ocular biometrics changed insignificantly when BMI increased. Unlike other biometrics, no significant tendency was observed in SER and CCT across quartiles of height, weight and BMI.

**Table 3:** Mean ocular biometry measurements and refraction by quartiles of height, weight and BMI

			Ocular biometrics (Mean ± SD)							
Stature	Range(Min ~ Max)	n	SER(D)	AL(mm)	CCT(mm)	ACD(mm)	LT(mm)	VCD(mm)	CR(mm)	AL-to-CR ratio
**Height(cm)**										
1st quartile	87.00 ~ 106.00	103	1.48 ± 0.75	21.90 ± 0.67	0.55 ± 0.03	3.44 ± 0.20	3.60 ± 0.14	14.32 ± 0.62	7.72 ± 0.24	2.84 ± 0.06
2nd quartile	106.50 ~ 110.00	105	1.24 ± 0.80	20.20 ± 23.35	0.48 ± 0.62	2.94 ± 3.96	3.25 ± 3.92	12.46 ± 15.75	7.25 ± 8.61	2.67 ± 3.04
3rd quartile	110.50 ~ 116.00	85	1.29 ± 0.77	22.39 ± 0.55	0.55 ± 0.03	3.53 ± 0.18	3.55 ± 0.13	14.76 ± 0.50	7.78 ± 0.20	2.88 ± 0.06
4th quartile	116.50 ~ 130.00	80	1.31 ± 0.86	22.54 ± 0.73	0.55 ± 0.03	3.52 ± 0.23	3.47 ± 0.14	15.01 ± 0.67	7.81 ± 0.23	2.89 ± 0.08
**P (trend)**			0.105	< 0.01	0.947	0.01	< 0.01	< 0.01	< 0.01	< 0.01
**Weight(kg)**										
1st quartile	11.50 ~ 17.00	136	1.38 ± 0.73	22.01 ± 0.68	0.55 ± 0.03	3.45 ± 0.20	3.56 ± 0.14	14.45 ± 0.65	7.72 ± 0.24	2.85 ± 0.06
2nd quartile	17.50 ~ 19.00	79	1.36 ± 0.83	22.18 ± 0.68	0.55 ± 0.03	3.46 ± 0.20	3.57 ± 0.14	14.59 ± 0.67	7.77 ± 0.23	2.86 ± 0.07
3rd quartile	19.20 ~ 20.50	66	1.31 ± 0.83	22.49 ± 0.56	0.56 ± 0.03	3.53 ± 0.16	3.54 ± 0.13	14.86 ± 0.54	7.82 ± 0.20	2.88 ± 0.06
4th quartile	21.00 ~ 27.50	92	1.24 ± 0.83	22.55 ± 0.63	0.54 ± 0.03	3.55 ± 0.23	3.48 ± 0.14	14.98 ± 0.56	7.81 ± 0.20	2.89 ± 0.07
**P (trend)**			0.139	< 0.01	0.668	< 0.01	< 0.01	< 0.01	< 0.01	< 0.01
**BMI(kg/m** ^**2**^ **)**										
1st quartile	8.06 ~ 14.37	94	1.38 ± 0.73	22.16 ± 0.71	0.54 ± 0.03	3.47 ± 0.20	3.53 ± 0.15	14.61 ± 0.69	7.73 ± 0.24	2.87 ± 0.07
2nd quartile	14.41 ~ 15.15	96	1.22 ± 0.81	22.33 ± 0.62	0.56 ± 0.03	3.51 ± 0.20	3.54 ± 0.13	14.72 ± 0.57	7.77 ± 0.22	2.88 ± 0.06
3rd quartile	15.18 ~ 16.16	90	1.38 ± 0.82	22.22 ± 0.72	0.55 ± 0.03	3.46 ± 0.22	3.54 ± 0.14	14.67 ± 0.67	7.79 ± 0.23	2.85 ± 0.07
4th quartile	13.00 ~ 27.50	93	1.35 ± 0.82	22.35 ± 0.70	0.55 ± 0.03	3.52 ± 0.20	3.55 ± 0.15	14.73 ± 0.67	7.80 ± 0.21	2.87 ± 0.07
**P (trend)**			0.875	0.155	0.263	0.431	0.235	0.318	0.013	0.049

P based on trend test

Table 4 shows the coefficients in linear regression models where the individual refraction or ocular biometric components as the dependent variables and height, weight, or BMI as the independent variables. In the univariable regression model, an increase in height and weight significantly affected ocular biometric growth, except for CCT. After adjusting for age and gender, both height and weight were significantly and positively associated with AL. The adjusted R2 of the model was approximately 0.28, indicating that 28% of the variance in AL could be attributed to difference in height or weight. Height and weight were also significantly correlated with VCD, with adjusted R2 values of 0.26. Although the correlation between height and weight with CR were significant, but only 7–8% of the variance of CR could be explained by the variance of height or weight, BMI showed marginal positive associations with CR, with an adjusted R2 of 0.07.

**Table 4:** Linear regression models of refraction and ocular biometry by height, weight, and BMI

	Univariable regression model			Multivariable regression model		
Stature	Regression coefficient (95%CI)	*P*-value	R2	(95%CI)	*P*-value	Adjusted R2
**Height(cm)**						
SER(D)	-0.0134(-0.0248,-0.0019)	0.022	0.0141	-0.0117(-0.0285,0.0051)	0.171	0.0358
AL (mm)	0.0354 (0.0261,0.0448)	< 0.01	0.1309	0.0199(0.0073,0.0325)	< 0.01	0.2762
CCT (mm)	0.00005(-0.0004,0.0005)	0.813	0.0002	-0.0003(-0.0010,0.0003)	0.355	0.0273
ACD (mm)	0.0054(0.0024,0.0084)	< 0.01	0.0334	0.0007(-0.0035,0.0049)	0.754	0.1166
LT (mm)	-0.0064(-0.0084,-0.0044)	< 0.01	0.0996	-0.0026(-0.0054,0.0003)	0.080	0.1273
VCD (mm)	0.0364(0.0277,0.0451)	< 0.01	0.1544	0.0221(0.0101,0.0342)	< 0.01	0.2616
CR(mm)	0.0038(0.0005,0.0072)	0.02	0.014	0.0050(0.0002,0.0097)	0.041	0.0721
AL-to-CR ratio	0.0030(0.0020,0.0039)	< 0.01	0.0951	0.0006(-0.0007,0.0020)	0.361	0.1659
**Weight (Kg)**						
SER(D)	-0.0208(-0.0475,0.0059)	0.126	0.0063	-0.0074 (-0.0390,0.0240)	0.641	0.0315
AL (mm)	0.0768(0.0549,0.0986)	< 0.01	0.1144	0.0406(0.0170,0.0641)	< 0.01	0.2797
CCT (mm)	0.0003(-0.0007,0.0013)	0.565	0.0009	-0.0001(-0.0014,0.0011)	0.828	0.0252
ACD (mm)	0.0133(0.0064,0.0201)	< 0.01	0.0377	0.0055(-0.0023,0.0133)	0.168	0.1209
LT (mm)	-0.0106(-0.0153,-0.0060)	< 0.01	0.0512	-0.0021(-0.0075,0.0032)	0.436	0.1215
VCD (mm)	0.0738(0.0533,0.0944)	< 0.01	0.1184	0.0373(0.0147,0.0599)	< 0.01	0.2567
CR(mm)	0.0125(0.0047,0.0202)	< 0.01	0.0274	0.0129(0.0039,0.0218)	< 0.01	0.0817
AL-to-CR ratio	0.0048(0.0025,0.0071)	< 0.01	0.0462	0.00003(-0.0025,0.0025)	0.998	0.1639
**BMI (kg/m** ^**2**^ **)**						
SER(D)	0.0117(-0.0396,0.0630)	0.653	0.0005	0.0147(-0.0359,0.0653)	0.568	0.0318
AL (mm)	0.0370(-0.0073,0.0813)	0.101	0.0072	0.0360(-0.0022,0.0742)	0.065	0.2642
CCT (mm)	0.0009(-0.0011,0.0029)	0.386	0.002	0.0007(-0.0012,0.0027)	0.465	0.0265
ACD (mm)	0.0107(-0.0027,0.0240)	0.118	0.0066	0.0102(-0.0024,0.0228)	0.111	0.1225
LT (mm)	0.0040(-0.0052,0.0132)	0.391	0.002	0.0026(-0.0059,0.0113)	0.543	0.1209
VCD (mm)	0.0215(-0.0205,0.0634)	0.315	0.0027	0.0224(-0.0144,0.0591)	0.232	0.2385
CR(mm)	0.0168(0.0019,0.0312)	0.027	0.0136	0.0147 (0.0002,0.0292)	0.047	0.0714
AL-to-CR ratio	-0.0020(-0.0065,0.0024)	0.368	0.0023	-0.0017(-0.0058,0.0024)	0.422	0.1654

All regression coefficients are derived from a separate regression model with the individual refraction or ocular biometric components as the dependent variable, and height, weight, or BMI as the independent variable. Adjusted by age and gender in the multivariable regression model

## Discussion

This study provides evidence on the association between body stature with ocular biometrics in preschoolers in southern China. We found taller and heavier preschool children had eyes with longer AL, deeper vitreous chamber, and flatter cornea. However, no association between body stature and refraction was observed when adjusted for age and gender.

As most of the juvenile myopia is axial myopia, AL elongation is considered the most crucial determinant of myopia incidence [15]. Previous studies have found that AL and height were associated and concomitant both in children and adults. After adjusting for age and gender, an increase of 10 cm in body height was associated with an elongation in AL for about 0.28 mm in Chinese adults in Singapore [7], 0.33 mm in young adults in northern China [6], and 0.27 mm in Australian children [11]. In our study, every increase of 10 cm in height results in 0.20 mm difference of AL elongation (the β between AL and height was 0.02 in the adjusted linear model), which was less than previous reports. The discrepancies in results among studies may be mainly attributed to the enrolled population. On one hand, axial length elongation may be mainly driven by physical growth in preschoolers, but more affected by environmental factors such as education and near work activities after entering schools [16]. The GUSTO birth cohort study has found no evidence of environmental factors influencing early-onset myopia. Taller children at birth and at 12, 24, and 36 months were found to have longer AL in 3 years old [17]. Another birth cohort study also reveals that taller and heavier neonates had longer AL at 6 years of age and growth during pregnancy and 2 years postnatally is the most important period underlying this association [18]. Thus, the relation between height and AL might be age-specific.

Associations between refraction and height were inconsistent in different studies [6, 7, 9–11, 19, 20]. Negative associations between refraction and stature i.e., taller and heavier persons with less hyperopic eyes, were observed in different ethnicities and regions [11, 19]. However, some studies found no association between height and refraction in preschoolers [17, 21, 22],which is consistent with our study. It can be easily understood that taller and heavier children have longer AL but flatter cornea, thus resulting in a slight difference in refraction in this study. Similar phenomenon has been observed that AL grows significantly with minimal changes in refraction in preschool children [23], suggesting body stature has limited potential in predicting refraction in preschoolers.

Similar to the previous studies [13, 18], taller and heavier children had deeper vitreous chambers and flatter corneas in our study. One possible explanation is that, not only AL elongation but also other ocular biometrics growth were concurrent with and driven by physical development in preschool children. Besides the genetic correlation between stature and AL [5], common determinant genes controlling body size and other ocular component dimensions such as cornea and lens were found in chickens [24]. The genetic height and birth weight risk scores were both significantly associated with ocular biometry in children [18]. Another possible reason is that the change of VCD and cornea are secondary to AL elongation. The secondary changes result from the physical stretching of eyeball as well as biological regulation to match optical power with the focal plane. Longitudinal and experimental evidence are needed to confirm these hypotheses.

AL-to-CR ratio was found to have a stronger association with refraction than AL in previous studies [25–27]. However, earlier reports of the association between body stature and AL-to-CR ratio were controversial in adults or school children [6]. A cross-sectional study conducted in 3-year-old preschoolers in China found no association between body stature and AL-to-CR ratio after adjusting for gender [28]. Similarly, we didn’t find a significant association between AL-to-CR ratio and height or weight in preschool children neither. On one hand, the AL-to-CR ratio in preschooler is relatively stable given the concurrent increase of AL and CR during emmetropization. Different ages, refractive error situations, and the lack of control for confounding variables may also result in conflicting results.

Reports on the relationship of BMI with ocular biometrics and refraction did not agree with each other. The Anyang University Students Eye Study found that BMI was significantly related to CR and ACD. Wu et al. found a strong association between BMI, AL, and CR after adjusting for age and gender [9]. But Wong et al. found that BMI was not related to those ocular biometric parameters [7]. We found no association between BMI and ocular biometrics and refraction in this study, indicating obesity may not influence the eye growth and refraction development. Supportive evidence has been found in the data from a total of 6855 ethnically-diverse Americans that body metrics nor nutritional factors (serum Vitamin D, glucose levels and caffeine intake) were associated with refractive error or myopia status [29].

The current study enrolled preschool children who were free from education pressure and mostly emmetropia, which enables us to investigate the direct relationship between body stature and ocular biometrics during emmetropization. Body statures have significant relationships with AL, VCD and CR, but not with refraction, revealing that taller and heavier preschool children have higher risk of myopia when compared to their emmetropia counterparts. Potential limitations of this study should be mentioned. Firstly, we did not include risk factors such as lifestyle, diet habits, environmental factors, and parental characteristics that would affect ocular biometrics development. Therefore, the impact of height and weight on ocular biometrics consists not only genetic effects but also underlying familial environmental influence. Secondly, the enrollment of preschoolers who were mostly emmetropia and less affected by education pressure enables us to investigate the impact of body stature on emmetropization process, but the small proportion of already myopic children may bias the results. Thirdly, this study is a cross-sectional study. The relationship between longitudinal change of body stature and ocular biometrics needs further investigations.

## Conclusion

In conclusion, stature parameters were associated with ocular biometrics in preschoolers but not with refraction. Height and weight both have a positive impact on the growth of axial length and vitreous chamber, whereas BMI does not. Height, weight and BMI do not influence CCT, ACD, LT and AL-to-CR ratio. However, they do have a significant but limited influence on CR. The growth of eyeball in preschoolers may be driven by physical development, whereas longitudinal shreds of evidence are still needed.

## Data Availability

The datasets used and analyzed during the current study are available from the corresponding author on reasonable request.
